# The neural circuitry of expertise: perceptual learning and social cognition

**DOI:** 10.3389/fnhum.2013.00852

**Published:** 2013-12-17

**Authors:** Michael Harré

**Affiliations:** Complex Systems Research Group, School of Civil Engineering, The University of SydneySydney, NSW, Australia

**Keywords:** expertise, perceptual template, theory of mind, social cognition, neural networks, stochastic differential equations

## Abstract

Amongst the most significant questions we are confronted with today include the integration of the brain's micro-circuitry, our ability to build the complex social networks that underpin society and how our society impacts on our ecological environment. In trying to unravel these issues one place to begin is at the level of the individual: to consider how we accumulate information about our environment, how this information leads to decisions and how our individual decisions in turn create our social environment. While this is an enormous task, we may already have at hand many of the tools we need. This article is intended to review some of the recent results in neuro-cognitive research and show how they can be extended to two very specific and interrelated types of expertise: perceptual expertise and social cognition. These two cognitive skills span a vast range of our genetic heritage. Perceptual expertise developed very early in our evolutionary history and is a highly developed part of all mammals' cognitive ability. On the other hand social cognition is most highly developed in humans in that we are able to maintain larger and more stable long term social connections with more behaviorally diverse individuals than any other species. To illustrate these ideas I will discuss board games as a toy model of social interactions as they include many of the relevant concepts: perceptual learning, decision-making, long term planning and understanding the mental states of other people. Using techniques that have been developed in mathematical psychology, I show that we can represent some of the key features of expertise using stochastic differential equations (SDEs). Such models demonstrate how an expert's long exposure to a particular context influences the information they accumulate in order to make a decision.These processes are not confined to board games, we are all experts in our daily lives through long exposure to the many regularities of daily tasks and social contexts.

## 1. Introduction

Those that have spent decades mastering a complex task have been the subject of considerable research and popular interest throughout the twentieth century and it is now a mature and well established area of research for the twenty-first century. Much of the interest in popular culture comes from the remarkable feats of accuracy, memory and speed these experts demonstrate with such relative ease when compared with the rest of us. It is hard not to be impressed by a chess Grand Master who can play against a half dozen other Grand Masters with little effect on the quality of their play (Gobet and Simon, 1996). Even more impressive is the ability of some chess players to play dozens of simultaneous games while *blindfolded* (Saariluoma, 1991). These are remarkable cognitive feats, but the basis of much current research is the idea that experts are not naturally or genetically *privileged*. There are aspects that are important; at what age training begins and the many hours of deliberate practice (Ericsson et al., 1993) play a vital role, but these are external factors. In principle at least, we all have the basic mechanisms that enable similar feats of excellence to be developed, up to some (possibly significant) degree of personal variation (Hambrick et al., 2013). On the other hand much of the scientific interest in this area stems from new ways in which neuro-imaging can be combined with expertise-specific tasks in order to analyze the basic neural processes that underpin these cognitive abilities and their development.

This article begins with the principle that there are universal mechanisms that exist in all of us but that experts have exploited these mechanisms to the very limits of their abilities. The significance of the universality of these mechanisms lies in the role “expertise” plays in all of our lives. For example we are all experts in facial recognition, we have a specific part of the brain dedicated to this task called the fusiform face area (Sergent et al., 1992). We are also experts in social reasoning, we have dedicated regions of the brain [such as the precuneus (Huth et al., 2012)] that enable us to perceive some situations as specifically social in nature. From this point of view, board games are a highly focused, complex task that involves multiply interacting processes, the constituent parts of which may be compared to what is already understood regarding simpler and comparatively well understood systems that may be differentially integrated and activated in experts.

This article addresses two specific aspects of expertise in board games using stochastic differential equations (SDEs), this is an approach to expertise that has not yet been explored in the literature but has been used extensively as a realistic model of neural dynamics in decision making and has lead to significant insights into the theoretical and computational modeling of neural dynamics (Bogacz et al., 2006; McMillen and Holmes, 2006). The first is the categorization of board positions by a fast feedforward mechanism and its integration with other neural processes as a source of *contextual information* (Harré et al., 2012). This framework captures the rapidity with which an expert can unconsciously appreciate a game's gestalt (Simon, 1986) and generate good options for the next move without conscious deliberation. The second is the ability of experts to understand the perspective of their opponent in deciding their next move. While strategic perspective taking is a little studied aspect of board game expertise there is considerable neuro-imaging evidence suggesting that perspective taking in economic games has mechanisms in common with board game expertise. With this in mind, the key contributions of this article are threefold: the introduction of an SDE formulation of expertise, its application to perceptual categorization in board games and its application to perspective taking in board games.

## 2. Perceptual categorization for experts

It has been hypothesized that expert perception in complex tasks is based on implicit learning of the statistical regularities of the environment to which the expertise pertains (Kahneman and Klein, 2009; Kellman and Garrigan, 2009; Harré, 2013). For example these statistical regularities allow a Grand Master chess player to rapidly categorize the current state of a game and generate good intuitive guesses as to what the next move might be. This requires a neural process of rapid consolidation of experience weighted percepts into a single categorical “whole” (Serre et al., 2007; Kriegeskorte et al., 2008; Wan et al., 2011; Harré and Snyder, 2012; Huth et al., 2012). An important implication is that for unconscious perceptual categorization of this sort there is no need for reward feedback, it is an unsupervised learning process as suggested by the early visual processing model in (Serre et al. (2007). Such processes have been the subject of psychological studies at least since the early work of Ratcliff (1978) on the brain's statistical accumulation of percepts to a decision boundary and Nosofsky's (1984) work on categorical similarity for decision making.

### 2.1. Stochastic processes as models of decision-making

This section introduces the mathematical framework in which perceptual decisions are modeled by SDEs. While the focus of this work is on binary decisions due to their simplicity of exposition, real decisions are the selection of one option from many alternatives. So while the work on stochastic decision boundaries (the basis of what follows) has recently been extended to multiple alternatives (McMillen and Holmes, 2006), the focus here is on the binary case. The simplest form of these equations is that of a time series of the incremental changes in a noisy variable *x* that has a constant (fixed) “drift” component μ and a statistical “diffusion” term σ dW (*d*W is a standard Wiener process with unit variance). During a time interval *dt* the change in *x* is given by:

(1) dx = μdt+σdW.

**Figure 1:**
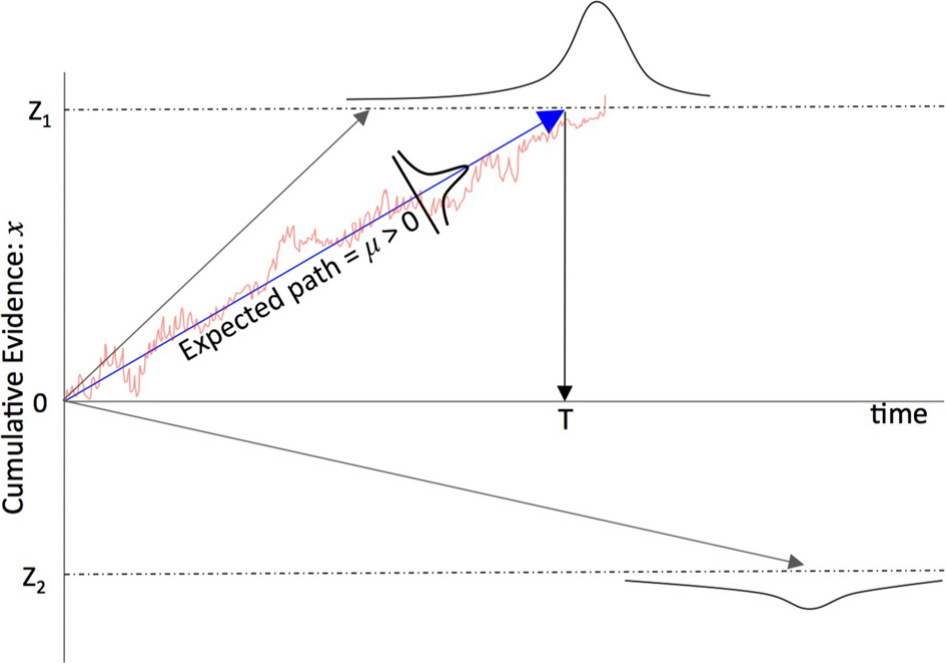
**A schematic of the net accumulation of evidence in favor of one choice over another and the mean trajectory followed by the “average” time course of evidence accumulation.** μ is the average amount of information received during *dt* over the time course of evidence accumulation. Note that because there is some statistical variation in the time course of the accumulation of information, for a non-zero σ (cf. Equations 1–4) there is a finite chance that the signal will cross the *wrong* boundary (and therefore the neuron signals the wrong decision) in the sense that the underlying signal is distorted to the extent that the boundary that is crossed is different from the boundary that would have been crossed in the absence of noise. So as σ increases so too does the chance that the wrong boundary is crossed.

With σ → 0 the noise term reduces to zero and *x* = μ*t* + *c* with integration constant *c*, i.e., a straight line with gradient μ and so the drift is thought of as the linear change in the accumulation of a signal *x*. With σ ≠ 0 the path followed by *x* fluctuates around the expected increase μ*t* with the fluctuations proportional to the variance σ^2^. In the perceptual decision literature this drift diffusion model is often interpreted as a threshold decision process: if *x* > Z_1_ or *x* < Z_2_ then *x* has crossed a decision boundary, *Z*_1_ or *Z*_2_, and a decision has been reached favoring one of two hypotheses, *H*_1_ or *H*_2_, represented by these two boundary values. This accumulation to a decision threshold is shown in Figure 1. A common interpretation, and the one adopted here, is that a neuron (or an assembly of neurons) can be thought of as a noisy accumulator of signals from other neurons such that when the neuron is excited to a level *Z* it fires thereby signaling a decision to those neurons to which it is (forward) connected. This is loosely described as a neuron's decision-making process or a neuron having “made a decision.”

There are three variations to this model that are interesting in the current context. The first is that the μ term can be split into as many components as needed to represent the dynamic microstructure within the interval *dt* providing these values are constants. For instance the composite term μ^*^ = μ_1_ − μ_2_ + μ_3_ is the “net drift” in *x* as the term μ^*^ is still constant, but the component parts μ_*i*_ may have neurological, psychological or perceptual interpretations that are useful to distinguish.

The second generalization is to split the process into two separate and independent stochastic variables: If μ_1_ and μ_2_ are independent stochastic processes with fluctuations σ_*i*_ then Equation (1) can be generalized:

(2) $$dx_{1}=\mu_{1}dt+\sigma_{1}dW_{1}$$

(3) $$dx_{2}=\mu_{2}dt+\sigma_{2}dW_{2}$$

This is often referred to as the race model: *x*_1_ races against *x*_2_ to reach their respective decision boundaries. In neurological modeling studies the *x*_*i*_ are seen as *independent accumulators*, they collect noisy signals from other neurons until they cross their respective decision boundaries. The average signals they receive are the μ_*i*_ plus fluctuations σ_*i*_dW_*i*_, so these are thought of as feedforward neural models, neurons that fire at a pervious time feed an average signal of μ_*i*_ that is aggregated in the decision variable *x*_*i*_ encoded by a neuron.

A third generalization is to introduce a dependency of the rate of change in *x* on the current state of *x*. In this case *dx* is driven by a constant drift term μ as well as the present state of *x*:

(4) dx=(μ+λx)dt+σdW

again noting that μ can be split into its (constant) constituent parts. This is called an Ornstein-Uhlenbeck (O-U) SDE, a common model of the neural processes involved in stochastic decisions (Bogacz et al., 2006). The interpretations of these models will be introduced as needed.

### 2.2. Stochastic decisions regarding category membership

These SDEs can be used as a model of neural interactions and category formation in the early stage, feedforward perceptual systems of the brain (DiCarlo et al., 2012). From this point of view a neuron receives signals from other neurons at an earlier stage of perceptual processing that encode a simpler set of percepts and this later neuron then aggregates these signals into a more complex representation. This is a process with some statistical variation due to the inherently noisy nature of the external environment as well as neural activity, but over a large population of neurons the information a higher level “scene categorization” neuron receives will represent the correct category. Figure 3A shows an abstract representation of the set of neurons that either excite or inhibit one of two higher level category neurons as modeled by Equations (2, 3). Figure 3B shows how two neurons (*E*_1_ and *I*_2_) can encode a signal that excites one categorical neuron while inhibiting the other.

The decision neurons *C*_1_ and *C*_2_ represent two different hypotheses regarding the state of the world. *H*_1_: the current scene can be represented as a category encoded by *C*_1_, *H*_2_: the current scene can be represented as a category encoded by *C*_2_. A decision regarding the state of the world is reached when evidence for one category accumulates to one boundary before an alternative boundary is crossed and so either *C*_1_ or *C*_2_ fires signaling the category for which there is more evidence. This process can be thought of as a stream of signals arriving at the retina and being processed by neurons that encode increasingly more and more complex representations that span an ever larger range of the visual field as the signals pass through a feedforward neural network (Serre et al., 2007).

An SDE representing changes in the level of excitation of neurons *C*_1_ and *C*_2_ is given by (see the caption of Figure 3 for the cell groups notation):

(5) $$\begin{array}{l} dx_{1}=[\overset{C_{1}\text{excitatory signals}}{\overset{︷}{E_{1}+E_{1}E_{2}+E_{1}I_{2}}}-\;(\overset{C_{1}\text{inhibitory signals}}{\overset{︷}{I_{1}+I_{1}E_{2}+I_{1}I_{2}}})]dt+\sigma_{1}dW_{1}\\ \;=\;\mu_{1}^{*}dt+\sigma_{1}dW_{1} \end{array}$$

(6) $$\begin{array}{l} dx_{2}=[\underset{C_{2}\text{ excitatory signals}}{\underset{︸}{E_{2}+E_{2}E_{1}+E_{2}I_{1}}}-\;(\underset{C_{2}\text{ inhibitory signal}}{\underset{︸}{I_{2}+I_{2}E_{1}+I_{2}I_{1}}})]dt+\sigma_{2}dW_{2}\\ \;=\mu_{2}^{*}dt+\sigma_{2}dW_{2} \end{array}$$

**Figure 2:**
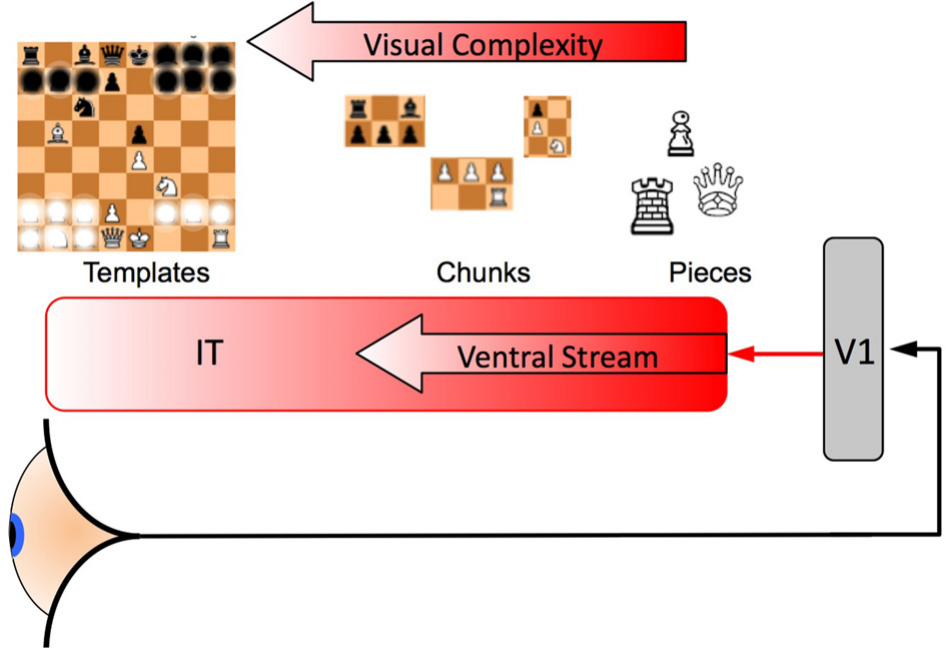
**A representation of the category formation mechanism.** Information arrives at the eye and passes to the anterior regions of the brain where these signals arrive at V1 (an early visual processing region of the cortex). These signals then pass along the ventral stream of the inferotemporal cortex (IT). Most people from a western country are likely to be able to recognize a single chess piece, more experienced players develop “Chunks” that are aggregates of games pieces as well as schematics for the whole board that include key pieces and their spatial relationships, these “Templates” enable the rapid comprehension of the current state of the game and provide “slots” into which the smaller chunks can be fitted [see for example Gobet et al. (2001) and Gobet and Lane (2010) who have modeled unsupervised template learning in their CHREST model and suggested a neural model for it (Chassy and Gobet, 2011), as does the perceptual templates in Harré (2013) and Harré and Snyder (2012)]. Some of the information (individual pieces) are shown obscured in the Template (far left), only information which is necessary to distinguish one category from the many other possibilities is needed, such game pieces need to have frequently co-occurred in the same places over and over again through a player's training and experience of the game. In this diagram only one template is shown, however, an expert will have encoded thousands of such templates in their IT and each one “competes” to be recognized: signals that originate from earlier neurons encode simpler representations (a line detected by V1 is simpler than a game piece, a game piece is simpler than a chunk and a chunk is simpler than a template) that combine to make larger and more complex representations of the game, eventually signaling the most complex learned representation such as a template. The neuron that accumulates enough (typically noisy) information to cross that neuron's threshold is the winner [see the race model, Equations (2) and (3)] i.e., for two categories the competition would be between neurons *C*_1_ and *C*_2_ in Figure 3 and the thresholds would be *Z*_1_ and *Z*_2_ in Figure 1.

**Figure 3:**
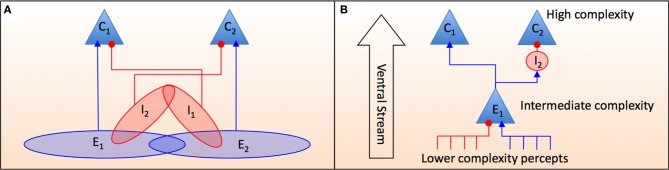
**A feedforward schematic of how percepts of intermediate complexity either excite or inhibit more complex percepts.** *C*_1_ and *C*_2_ are neurons that encode high order scene categories that are composed of percepts of an intermediate complexity. **(A)** A conceptual framework of cell assemblies that act to excite and/or inhibit more complex categorical representations. The groups of neurons *E*_1_, *E*_2_, *I*_1_, and *I*_2_ are each a set of neurons some of which have overlapping functionality between excitation and inhibition. For example the cells in the group *E*_1_*I*_2_ = *E*_1_ ∩ *I*_2_ excite *C*_1_ and inhibit *C*_2_ whereas the cells in group *I*_1_*I*_2_ = *I*_1_ ∩ *I*_2_ inhibit both *C*_1_ and *C*_2_. **(B)** A more detailed model showing how a neuron in assembly *E*_1_ receives noisy inhibitory and excitatory signals from other percepts. It can then directly excite percept *C*_1_ while indirectly inhibiting *C*_2_ via the excitation of intermediary inhibitory neurons that terminate on *C*_2_. The “Ventral Stream” is the feedforward direction of the neural signaling as shown in Figure 4.

Note the similarities of Equations (5) and (6) to Equations (2) and (3) and that the μ^*^_i_ are simply constants independent of the *x*_*i*_ (also note the discussion regarding μ^*^ just before these equations). In Equations (5) and (6) the μ^*^_i_ terms represent the deterministic component of the net drift of the stochastic variable *x*_*i*_ toward a threshold value *Z*, this threshold represents the evidence necessary to recognize a category. This drift in *dx*_*i*_ is an aggregation of visual signals and such aggregation of signals is not perfect, so some signal (such as the detection of a “spikey hat” on the top of a chess piece) might suggest a Queen piece or a King piece when in fact it is a bishop, however, when aggregated over many different visual cues we often accurately differentiate Kings from Queens from Bishops. These stochastic infelicities occur at all levels of the processing of visual signals, so we model these noisy signals using SDEs just as is done in studies of simple perceptual decision making, the key difference here lying principally in how far along the perceptual hierarchy the neurons in question happen to lie, this perceptual hierarchy runs along the ventral stream from right to left in Figure 2 and from top to bottom in Figure 3. So in order to categorize a whole board, a bishop in a certain position might inhibit the recognition of a certain game opening (because it never occurs in that position for that particular opening) while it excites the recognition of an alternative opening, but such a recognition is again imperfect, the square on which the bishop is placed may be misidentified by the player or a critical nearby pawn might be overlooked. Some of these infelicities might be addressed through slower and more deliberate analysis of the board position, but this may not always be effective as the perception of the game category can set the context on which further deliberate analysis of the board is based.

The visual processing of scene information goes from the perception of simple lines and angles in region V1 through to actual objects such as single chess pieces and ultimately to a representation of the category to which the board configuration belongs, if the player is experienced enough to have learned such categorical representations, see Figure 2 that summarizes some of the key ideas in the literature. This is different from recognizing every individual game element on the board, a *category* in the current sense means the broad strategic layout of the game, usually indicated by a number of key game pieces in particular key positions (Harré and Snyder, 2012; Harré, 2013). So minor variations on a particular chess opening can belong to a single category, indicated by key pieces in key positions that have frequently occurred in these same positions. This is what is meant by the statistical regularity of the environment, it enables a chess player to implicitly learn the strategic context of a game and then, as they grow in experience, to use this context in their search for good moves based on the implicitly recognized cues involving spatial relationships, color and some pieces. But an expert player will have acquired many thousands of different such categories over their career so the *i* in *dx*i of Equations (5) and (6) runs into the 1000's. Recent work has extracted these templates and enumerated them using an artificial neural network and real games of amateurs and professionals (Harré, 2013). And just as identifying a single game piece can be a statistically uncertain process, so too is identifying the current game's categorical membership out of the many thousands of possibilities.

These SDEs are similar in nature to those described in Bogacz et al. (2006) as well as the hierarchical structure recently proposed by Serre et al. (2007) and DiCarlo et al. (2012) where feedforward inhibitory and excitatory signals compete to accumulate evidence for one category over another. The key notion of this new approach is the use of SDEs to describe the neural mechanisms and to apply these ideas to expertise. So in this model a visual signal will elicit a combination of signals from neurons in precursor neural assemblies (of intermediate complexity) *E*_1_, *E*_2_, *I*_1_, and *I*_2_ (Equations 5, 6). The solutions to these equations can be usefully expressed in terms of the probability that one of the category boundaries is crossed (Bogacz et al., 2006):

(7) $$p(Z_{j}\text{ is crossed})=\frac{e^{\beta \mu_{j}^{*}}}{\sum_{k}e^{\beta \mu_{k}^{*}}}\;j,k\in \{1,\ldots ,1000\text{'}s\}$$

where β = 2z/σ^2^ (for simplicity symmetrical decision boundaries are used: *j* ∈ {1,2}, Z_1_ = Z_2_ = *z*), see Figure 1 for a schematic of the binary categorization dynamics and the probability of crossing one threshold versus another. Equation (7) has a very simple interpretation: The probability of recognizing “board category” *j* is a function of the sum of the evidence μ^*^_j_ in favor of that category (relative to the evidence for other categories μ^*^_k_) subject to some statistical variability parameterized by β.

## 3. Perspective taking and our “strategic theory of mind”

The previous section extended the well studied modeling paradigm of SDEs to the issue of expert board perception and rapid categorization. While this is a novel extension of recent work the goal is relatively modest in that it aims to connect the theoretical principles of two approaches to the modeling of both simple and complex perceptual decisions.

This next section has a more ambitious goal: to use an extension of these SDEs (a hybrid combining Equations 2, 3 with Equation 4) as a model of decision-making processes whereby the decision-maker has an internal representation of the perspective of another person such as a chess opponent. One of the assumptions made in what follows is that in order to understand another person's perspective an individual needs a representation of the other's internal mental states e.g., their constraints and goals, and that these might be different from those of the first person. At this point two concerns arise: *Is this a reasonable assumption?* and *What is the evidence for such an assumption?* The latter will be covered in sections 3.1–3.3 but a few words are needed first to justify the reasonableness of this approach.

**Figure 4:**
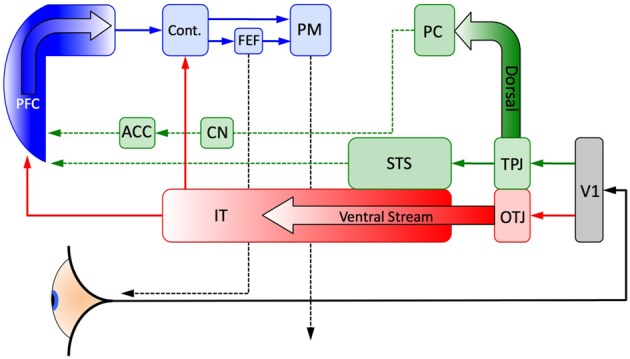
**A schematic representation of the major cortical pathways of expertise: feedforward visual processing (red), strategic Theory of Mind and associated reward mechanisms (green) and top down planning and control (blue) discussed in the main text.** The anterior-most frontal regions integrate information from a strategic ToM network and a perceptual network for recognizing individual items of intermediate complexity. The categorical signal projects to a region posterior to the anterior regions of the frontal cortex that are associated with high level strategic planning and control processes. This allows for the possibility of the contextual signal to rapidly activate an eye saccade (and other motor activities) to a strategically relevant portion of the visual field without passing through the top–down planning areas in the PFC. Such search guided by implicitly learned visual cues was established by Chun and Jiang (1998, 1999) for large but relatively simple environments. V1, visual area 1 in the occipital cortex; OTJ, occipitotemporal junction; TPJ, temporoparietal junction; STS, superior temporal sulcus; IT, inferotemporal cortex; PC, precuneus; CN, caudate nucleus; ACC, anterior cingulate cortex; PFC, prefrontal cortex; Cont., visual context integration; FEF, frontal eye field; PM, primary motor cortex.

When a skilled player looks at a game in progress there may be sufficient information available in the first few moments of viewing the game for the player to make a decision as to where to move their eyes in order to refine their search such that only the most promising areas of the board are explored (De Groot et al., 1996). This fast perceptual comprehension is an unconscious aspect of expertise and it is an important part of an expert's remarkable speed in selecting a good move from a very short exposure to a game position (de Groot and de Groot, 1978). The idea is that for exceptionally familiar positions the next move is so well understood that very little (if any) further analysis is necessary in order to know what the best move is. In such situations no comprehension of the other player's mental state is necessary, purely perceptual processes based on their extensive experience are sufficient to explain an expert's behavior and performance. If this is the case then very little planning and control beyond the early stages of perception is needed and a player's decision-making can circumvent the slow and computationally expensive sequential process of searching multiple alternative branches of play in order to find the best strategy and instead can move directly to organizing the motor pathways necessary to physically move the player's arm to make the move on the board. In Figure 4 this is shown as the frontal region of the inferotemporal cortex providing a contextual signal directly to the primary motor area, i.e., a contextual signal generated by the activation of a single Template (see Figure 2) can contain sufficiently unambiguous information on which to base the next move. Also in Figure 4 can be seen how such a contextual signal might connect directly to the frontal eye fields in order to signal the eyes of an expert [an expert's Templates guide their visual search Chun and Jiang, 1998] to quickly orientate their eyes to relevant regions of the board.

Even for very experienced players skilled perception is often necessary but it is not always sufficient for strong play, in such cases the state of the game will have information that enables a player to refine their choices but still leaves ambiguous exactly which move to make. So templates need to be supplemented with more deliberate strategic planning and analysis. Such *executive control* for any task is thought to occur in the prefrontal cortex (PFC, Figure 4) (Koechlin and Summerfield, 2007). The PFC integrates information from diverse regions of the brain (Miller and Cohen, 2001), this is part of a bottom–up process but note that the PFC can be circumvented for rapid and automatic behaviors (Miller and Cohen, 2001). The PFC also exerts top–down control, for example modulating earlier perceptual signals (Bar et al., 2006) and even directly eliciting long term memories stored in the inferotemporal cortex (IT) (Tomita et al., 1999) (Figure 4 only shows feedforward signals to the PFC, but feedback pathways exist from the PFC to the IT). From this point of view the PFC modulates and integrates perceptual signals with internally generated goals, plans future actions and acts as an informational switchboard for other regions of the brain. So it is in the PFC that we should expect planning, strategic analysis and forward search to be carried out for complex tasks such as chess (see sections 3.1–3.3 for a selection of the literature supporting this), which might include a representation of another player's state of mind.

In terms of chess playing, when a player is planning their next sequence of moves, each player will associate a different “value” or place different constraints on the same move, for example a Black Kingside castling is never a move that white can make, so the constraint on this move is “illegal” for white but “legal” for black. A more sophisticated example is how a player represents the motivations on which another player bases their decisions. To illustrate this imagine a very well known chess opening has been played out for the fist three moves by each player. How does player 1 consider their choice of next move given that it is only a “good” move within the context of what player 2 might do in reply? It is not sufficient for player 1 to have a singular value of a move, a move's value is contextualized by the other player's likely next move, and player 2's likely next move is based on how player 2 values their move within the context of what player 1 will do following player 2's move. From this point of view player 1 needs to encode their estimate of the value of a move as well as the value player 2 will attach to the move player 2 is likely to make next. This regressive process of player 1 evaluating their choices in the context of player 2's likely choices is also the basis of economic game theory and requires each player to be able to approximate the other's mental strategic space which can include the other player's evaluation of the game, the strategy they seem to be following as well as the other's experience and habits, should these be known to player 1. These cognitive processes of the PFC are informed by the early perceptual signals the PFC receives from regions such as the IT but they are also based on a player's internal representation of their own strategy and how this strategy is contextualized by their internal representation of the other player's strategy.

With these ideas in mind, research into our “Theory of Mind” (ToM) focuses on the psychological and neurological mechanisms through which we understand the internal mental states and goals of other people (Lieberman, 2007) but it has not previously been connected with board games and expertise, and only in simple economic games such as those used in neuro-economics have SDEs been used to model these simple choices. ToM research covers a very broad range of topics, from psychological and neurological development through to genetic differences, traumatic brain injury and neuro-degenerative diseases. This breadth is due at least in part to the extensive interrelated cognitive processes that are involved and the very deep connection that our ToM has to the way in which we introspectively view ourselves, others and the choices we individually and collectively make.

In this section the goal is to first emphasize the overlapping neurological processes that play a role during ToM processing tasks, economic games (simple games), and board games (complex games). An important caveat is that the resultant model represents a strict subset of neurological processes that will be called our *strategic ToM*. Having illustrated the plausibility of a common neural mechanism, an SDE model of strategic social interactions will be introduced. The significant components are the cognitive ability to separate rewards received in the first person versus rewards received in the second person and a conscious perception of the relevant components in the external environment.

### 3.1. Neuro-economic game theory and our “strategic IQ”

The term *strategic IQ* was introduced in Bhatt and Camerer (2005) where the neural correlates of self-referential strategic reasoning, i.e., reasoning about someone else reasoning about you, in economic games were demonstrated using fMRI analysis. As discussed above, this is a minimal cognitive ability necessary to understand our actions in the context of other people's actions, but it is not the only necessary mechanism. In this section a sample of the fMRI literature on game theory, strategic IQ and ToM is explored and in the section that follows our strategic ToM is introduced and the literature from board game expertise supporting such an extension is surveyed.

A key point of interest in neuro-economics involves the neural regions that are active when we are thinking about our decisions in the context of other people's decisions. This entails at least some of the mechanisms that are active during ToM tasks and so there is an overlap between ToM research and neuro-economics. This has recently lead Yoshida et al to proposes a *game ToM* (Yoshida et al., 2008) using the simpler economic games to motivate their basis of a ToM. ToM studies include a broadly defined and general purpose network that is activated in many situations in which inferences need to be made regarding the cognitive states of others. But there is also a specific sub-network that is activated in strategic interactions such as economic games where assessing another person's internal states is necessary for our performance in strategic decision making. As such accurate predictions regarding how others think about their environment as well as how they think about us improves our outcome in the interaction. This second definition narrows our focus, the ToM networks of neuro-economics are concerned with strategies and expected rewards and this circumscribes the situations considered.

The broad definition of a ToM neural network frequently includes (Amodio and Frith, 2006) the medial prefrontal cortex (mPFC), the temporal pole, the superior temporal sulcus (STS), the anterior cingulate cortex (ACC) and the temporoparietal junction (TPJ). Many of these areas may or may not be active during strategic interactions as they might play a role in more general social cognition. The TPJ for example appears to be active in many different social contexts such as when a person is simply observing other people interacting (Saxe and Kanwisher, 2003).

Using a combination of results from game theory and fMRI studies a related network of neural activity can be identified. This article is not a review of this entire field, but there are two specific types of task to focus on. In the first type a subject plays a game against a computer or a human and differentiated neural activity shows brain regions that are active when we play strategically against another socially aware subject. This enables us to differentiate between “social” and “non-social” strategic interactions and the associated brain activations. The second type is one in which subjects play games that involve different levels of strategic thinking regarding their opponent's thought processes. In this second task, a key finding is the correlation between the reward earned and increases in brain activity in specific areas.

When playing strategic games against a human as opposed to a computer the brain regions that are differentially activated include the ACC (McCabe et al., 2001; Gallagher et al., 2002; Sanfey et al., 2003), the STS (Rilling et al., 2004; Fukui et al., 2006; Coricelli and Nagel, 2009), the TPJ (Krueger et al., 2008; Coricelli and Nagel, 2009; Carter et al., 2012), the mPFC (McCabe et al., 2001; Bhatt and Camerer, 2005; Coricelli and Nagel, 2009) and the caudate nucleus (CN) (Bhatt and Camerer, 2005; Delgado et al., 2005; Rilling et al., 2008). As these regions are differentially more active for human opponents than computer opponents it suggests that these brain regions play a role in social competitive situations. This does not preclude them from playing a role in other strategic and/or social tasks of course.

On the other hand a player's strategic IQ is correlated with a related network of brain regions. In one recent study stronger activity in the precuneus and the CN (Bhatt and Camerer, 2005) correlate with strategic IQ in games that differentiated between degrees of belief regarding the other player's strategy. In a similar study it was shown that the depth of interpersonal strategic reasoning co-varied with activity in the medial PFC (Coricelli and Nagel, 2009). In a third study, along with the reward prediction based activity of the medial PFC it was shown that activity in the posterior STS was strongly correlated with the influence a player's action's had on another player (Hampton et al., 2008). These studies identify a network of key brain regions that are active in strategic situations involving economic rewards: the mPFC, the CN, the ACC, the posterior STS and the PC, all of which are strongly related to the strategic success of a player and that have an overlap with those regions that also play a role in our ToM network.

### 3.2. Perception, games and a strategic theory of mind

Board game expertise activates a large neural network with many interacting brain regions that can be differentiated on the basis of the task involved. In recent work it has been shown that the neural networks activated by game experts involves a large number of brain areas, some belong to the visual system and some to the ToM system, but these findings have not yet been integrated in terms of the overlaps and differences possibly due to the different research areas to which they belong. This section discusses four particular articles that have recently shed significant light on the different brain regions involved in board game expertise. These results are discussed in terms of a single system for board game expertise that encompasses both ToM and visual perception.

In two recent fMRI studies (Bilalić et al., 2010, 2012), Bilalić et al have explicated the brain regions that are activated in expert chess play and their relationship to rapid eye movement toward areas of strategic importance. This is a purely perceptual body of work and so usefully isolates the perceptual mechanisms that generate eye movements without the need for considering the social context in which games are played (cf. the introductory remarks to section 3). Specifically they identified the ventral visual path (in the temporal cortex) as playing an important role in recognizing game pieces as well as familiar positional relationships between the game pieces in support of the role the IT plays in generating eye movement signals (cf. Figure 4). In the dorsal visual path the region forming a conjunction with the parietal, occipital and temporal cortices was found to be related to specific game pieces and their functional roles. A further activation in the retrosplenial cortex was also observed, a region that has been identified with scene context and the authors suggested this region plays a role in parsing the relationships between objects. Beyond the neurological findings, both of these studies highlighted the differences in eye movement between novices and experts. Experts focused quickly on the task relevant pieces in the scene and ignored irrelevant pieces whereas novices attended to irrelevant pieces much more often. In control tasks in which game piece relationships could not be used to guide the expert's behavior their performance decreased significantly but still maintained an advantage over novices.

A third article by Wan et al. (2011) considered three ranks of players, low ranked amateurs, high ranked amateurs and professionals of the Japanese board game Shogi. The players were required to generate the next move as quickly as possible while fMRI brain imaging followed the time-course of neural activity. The PC and CN were two regions that were strongly activated in professionals but not amateurs. As previous studies have shown that the PC is activated in understanding social contexts (Huth et al., 2012) and the CN is activated in strategic interactions with other people (Delgado et al., 2005; Rilling et al., 2008) as well as correlating with depth of strategic reasoning (Bhatt and Camerer, 2005), this suggests that the results of Wan et al. (2011) overlap with strategic reasoning, and as argued above strategic reasoning is a subset of the cognitive processes used during some ToM tasks. There was also considerable activity in the dorsolateral PFC for both amateurs and professionals when contrasts were made with control tasks. The authors concluded in part that this was not a direct “stimulus-response” activation as the players reported being unable to figure out a complete strategy before making their next move selection. Instead they concluded that “the generation of the next best move had to be based on perception of key features extracted from the pattern but not the pattern itself. In other words the mapping from inputs to outputs had to be categorical.” Furthermore, the players were not able to picture all of the necessary intermediary moves required to complete the checkmate task they were given, instead they were only able to “get an idea of the arrangement of key pieces at the final checkmate.” Beyond the activation of the PC and CN, an indicator of a socio-neural response, the conclusion that can be drawn is that categorical recognition and pattern completion are two key aspects of an expert's ability to quickly generate the next move in board games.

The fourth study was on the role of expertise in board games (Duan et al., 2012) in combination with the “default mode network” (DMN) (Raichle et al., 2001). The DMN is the resting activity of the brain and it plays a significant role in our understanding of ToM (Spreng et al., 2009). What is most interesting about this network is that it is significantly deactivated during goal directed tasks, presumably so that our cognitive functions can focus on the external environment rather than internal, reflective or introspective ruminations. In the study by Duan et al. (2012) Masters and Grand Masters (experts) of the game Chinese chess were imaged using fMRI for their resting state neural activity and for their task induced (Chinese chess problem solving) neural activity. There were two key findings: experts significantly deactivated the DMN relative to novices and the CN was considerably more active in the expert's DMN than that of the novice's. This should be contrasted with the areas commonly associated with ToM, in the earlier list of ToM brain regions the CN was excluded, this finding by Duan et al suggests that the DMN network (and therefore the ToM network) is significantly different for experts.

The relevance of these findings is in the relationship between the neural networks activated during economic games, during expert task execution and our understanding of another person's cognitive state. With this in mind the CN plays a striking role: it is significantly active in the DMN for experts, board game experts and in strategic economic games. The role the CN is commonly attributed with is in relation to feedback based learning (Haruno et al., 2004) so it is not so surprising that it should be active in economic games when rewards are earned based on performance, and perhaps even in the case of board games where rewards tend to be more abstract (winning or losing after many moves are made as opposed to money received immediately). But its role in the DMN is not immediately intuitive, but it might be understood to play a role in the encoding of another's payoffs as well as personal payoffs. Three recent papers have shown that this is a plausible role for CN; the macaque monkey CN encodes its own rewards as well as social status (Santos et al., 2012), in humans the CN is active in cooperation between people where no rewards are forthcoming (Krill and Platek, 2012) and it also encodes another's “moral character” in economic games (Delgado et al., 2005). These studies point to the CN as potentially encoding value judgements regarding other players as well as our own. Such estimates of another person's evaluation of the situation seems a likely minimum for estimating the motivations for another person's choices, and so representing another person's estimated value in order to model their motivations may well co-opt the pre-existing system of reward feedbacks that play a critical role in motivating our own decisions in a non-social context.

Strategic IQ is a measure of the depth to which we are able to reason about other people thinking about us thinking about them thinking about us etc. as measured by payoffs in economic games (Coricelli and Nagel, 2009). This is potentially an infinite regress for which there is no stopping point, however, it has been shown that an *equilibrium point* can be reached in this dynamic, and this equilibrium has been measured in the neural activity of people playing economic games (Bhatt and Camerer, 2005), but to date no theoretical model of the neural mechanisms involved has been proposed. On the other hand, a ToM is a very general cognitive process (Lieberman, 2007) that enables people to build a representation of the cognitive states of another person, including their beliefs, constraints, perceptions and potentially a ToM allows one person to build an internal representation of another person's representation of them. So the definitions of ToM and strategic IQ have commonalities but they are either too broad (ToM) or too narrow (strategic IQ) to capture the processes that are likely being used by decision-makers in complex, social-competitive situations. To address this the term Strategic ToM encompasses the psychological aspects, neural dynamics and subsequent equilibrium points necessary to finitely represent our representation of another person thinking about us, but expanding on the strategic IQ notion to include the ToM components important to complex social-competitive decision-making.

Taken collectively, these studies have identified a network of activity that encodes visual perceptual cues and task specific objects as well as reward and social learning mechanisms in combination with aspects of a strategic ToM. Some of the most commonly cited and important brain regions in this network are identified in Figure 4 for the perceptual aspects (red) and the strategic ToM aspects (green). This is necessarily a reduction to only the simplest functional roles and relationships, but it gives an indication of how these regions likely combine together to form a multifunctional network of interrelated brain regions. In the next section a theoretical model of rewards and strategic ToM is introduced, providing a theoretical approach to understanding some of the mechanisms discussed in this section.

### 3.3. An example of plausible neural activations during game play

**Figure 5:**
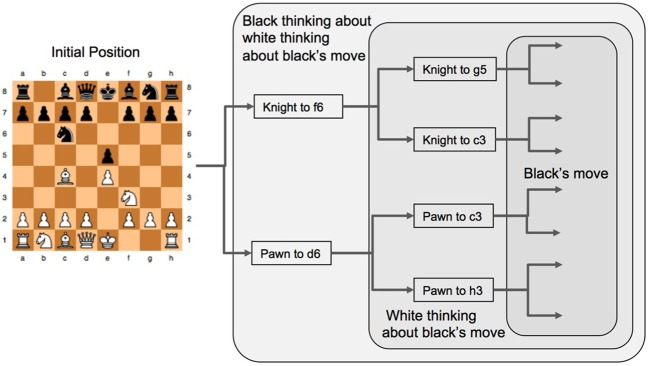
**The game tree for forward search in a game.** Black is to play first from the initial position and the tree analysis is simplified to considering only two possible branches at each stage. Knight to *f*6 and Pawn to *d*6 are two different “base moves” from which black then begins their analysis of subsequent play. In order for black to estimate the success of these two base moves they need to consider how White will reply, and the way White will reply depends on how white evaluates how black will reply to White's moves, hence Black needs to evaluate how white will evaluate Black's reply to White's next move. Note that Black's final move choices shown here (pawn to *d*5 and Bishop to *c*5) are the same irrespective of what White chooses prior to these moves, this is a strategy that might occur in a real game. However, white values Black's possible responses differently depending on what move White is considering (either the Knight or the pawn) and subsequently this changes what Black might prefer to do when choosing either the Knight or the pawn as their very first move in this sequence. Such considerations in complex games have some commonality with economic games.

Before introducing the SDE dynamics of a strategic ToM we want to motivate what follows by illustrating the ideas presented so far with a worked example. To begin, a chess game opening is already in progress (as shown in Figure 5) and we ask what are the brain regions that might be activated in seeing this game and what roles do they play? We use the simplifying assumptions that most of the signals we are interested in will first pass through region V1 so that we are only considering the visual aspects of the game and that the players only ever consider two moves as they search forwards in the game looking for good moves to make. This second simplification makes the discussion much simpler, but the ideas can be readily extended to multiple base moves and multiple subsequent branches.

The social and reward related neural networks (green path in Figure 4) are activated when the player first sees the game. Generally the TPJ is activated if the situation requires understanding the internal mental states of another person (Saxe and Kanwisher, 2003), it is activated when strategically thinking about other people rather than computers (Krueger et al., 2008), its activation level correlates with the depth of strategic reasoning (Coricelli and Nagel, 2009) and it is associated with socially guided decisions (Carter et al., 2012). The PC is activated if the current situation is a social context involving people, movement, certain animals, cars, tools, equipment, talking etc. (Huth et al., 2012) as well as ToM tasks (Saxe and Kanwisher, 2003), but in this case it happens to be a chess game in progress (Wan et al., 2011), perceived visually by the chess board, game pieces and another player. The CN activation is strongly associated with activation of the PC in board games (Wan et al., 2011) and with the ACC when learning (reward feedback) in economic games (Sanfey, 2007) and in distinguishing between “me” and “not me” based rewards (Tomlin et al., 2006). Finally in this social/reward path the STS is activated during ToM tasks (Saxe and Kanwisher, 2003) and in the perception of intentional behavior in other people (Gallagher and Frith, 2003). Taken as a combination of activations, this neural network recognizes the social context of the board game and the tools of this social context (the board and chess pieces). It also recognizes that another person is involved in the situation and that they have internal mental states that will play a role in the decisions that will need to be made. Finally the feedback from the outcome needs to distinguish between rewards received by the first player (“me”) and the second player (“not me”) for their respective choices in order to accurately attribute each player's gain or loss to the relevant player and strategy, see section 3.4 for further discussion about each player's rewards and their influence on choices. This establishes the immediate social context in which decisions will be made.

The visual perception of this opening position is also activated when first seeing the game. The occipito-temporal junction (OTJ) is differentially activated for chess experts when compared to both controls and non-expert chess players as well as playing a role in guiding the eye movements of experts when searching the board for their next move (Bilalić et al., 2010, 2012). The ventral stream along the inferotemporal cortex as a whole has been extensively studied in humans and other primates (Kriegeskorte et al., 2008) and it has been computationally modeled in terms of more and more complex visual representations of larger and larger portions of the visual scene (Serre et al., 2007). So this ventral pathway identifies visual objects such as individual chess pieces and constructs progressively more complex representations of these objects, including their familiar spatial relationships. As has been suggested in Wan et al. (2011) sufficiently complex representations of a board game are categorical representations, it is not an exact pattern matching process. Once the ventral path has aggregated the visual scene to the extent the player's experience makes this possible, a “context” signal can follow one of two paths. If there is sufficient information in the contextual signal to suggest a single move then the primary motor area (PM, Figure 4) is signalled to make that move. Alternatively a signal arrives at the frontal eye field (FEF, Figure 4) to tell the eyes where to move to next in order to explore different regions of the board, this is the basis of expertise guided search based on the contextual cues embedded in scenes (Chun and Jiang, 1998) and games (Bilalić et al., 2012). This establishes the initial perceptual processing of the board as a visual “scene.”

The final process shown in Figure 4 is the activation of the PFC and the subsequent processes that lead to the player actually making a move on the board (blue path). In the chess example this is where the social context, the differentiated roles of the two players and the perceptual information are integrated so that a coherent strategy can be developed. If a move has not yet been made (i.e., the context was not sufficient to suggest a move to make immediately) then the eyes are searching the scene providing more information to the PFC. This information needs to be integrated in terms the constraints, plans, goals and incentives of the player as well as a representation of the same mental states of their opponent. With this in mind Koechlin et al. (2003) and Koechlin and Summerfield (2007) have proposed a model of cascading levels of processing that begins in the most anterior regions of the PFC and ends at the posterior region of the PFC just before the PM cortex, this is the anterior to posterior path shown in blue of Figure 4. Importantly, the most anterior regions of the PFC seem to play a role in the integration of the outcome of multiple cognitive processes when a person is pursing a higher behavioral goal (Ramnani and Owen, 2004). In terms of exploring and planning possible moves in chess, the eyes foveate a potentially useful region of the board, this is called a “base move” (Gobet, 1997), guided by perceptual templates and from this region branching strategies of potential moves the player and their opponent might make are then searched to find a good intermediate position in the game (either a piece captured or a strong strategic configuration). So in Figure 5 the Black player's eyes initially saccade to the pawn at *d*7 and the player considers moving this pawn to *d*6 from which a number of alternatives are possible for White to then play and Black to then reply etc., two of White's options are shown in Figure 5. The black player's eyes then saccade to the knight at *g*8 and considers the sequence of moves that begins with a move of the knight to *f*6 and a sequence of possible plays is shown in Figure 5. Once the black player has decided which of these two strategies to adopt (knight to *f*6 or pawn to *d*6), the neural activation *cascades* (Koechlin and Summerfield, 2007) from the anterior regions of the PFC in which strategic planning and branching has been mapped out at the conceptual level to the posterior regions of the PFC where a motor plan leads to the moving of the relevant parts of the body (Ramnani and Owen, 2004) to shift a game piece in order to make the first move. While this style of reasoning is somewhat similar to that used in the much simpler (and strategically different) economic games (Bhatt and Camerer, 2005), it is far more complex in that perception, constraints, learning and uncertainty in evaluations play significant roles in the decision-making process over and above the purely strategic structure of the game and the payoffs of economic theory.

### 3.4. Stochastic dynamics for a strategic theory of mind

**Figure 6:**
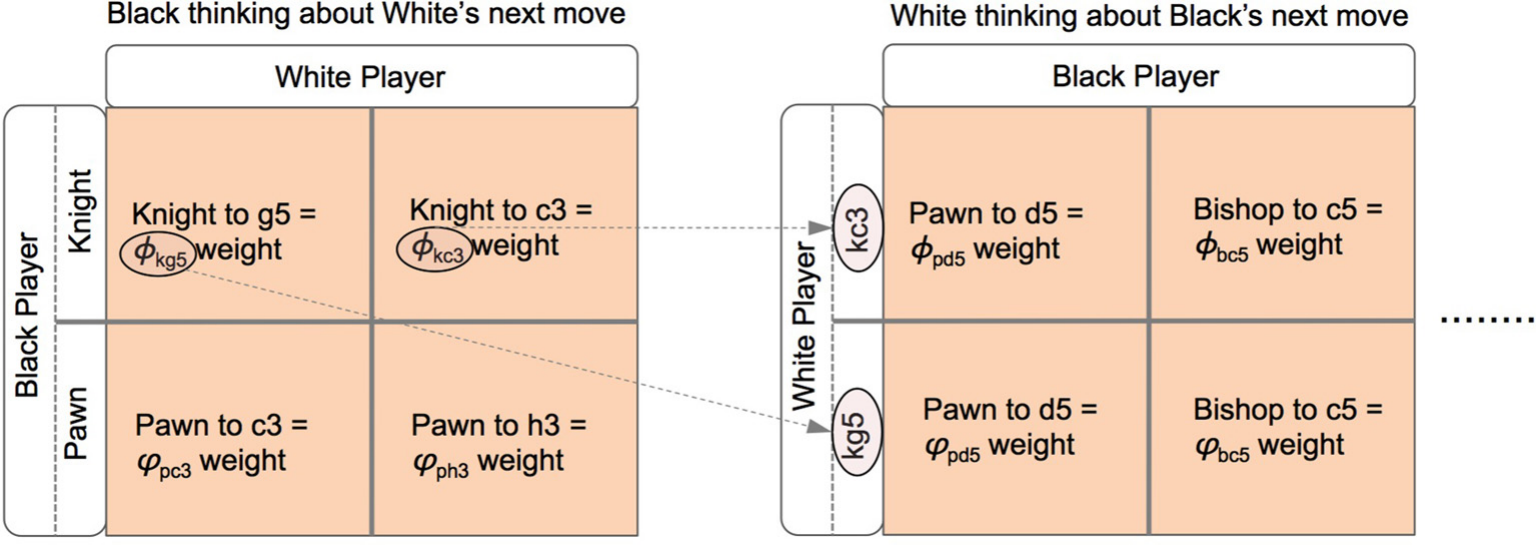
**A matrix representation of the estimated weights that Black uses to analyze their next move (left) and how White will respond to each of these moves and the rational that White will use to evaluate their subsequent choices given Black's prior choices (right).** In order for Black to understand White's preferences for their next move, Black needs to encode a representation of the way in which White will select their move. This is the same process as Black uses to make their decision but using information pertinent to White's strategic position.

In order to model one player's internal representation of another player, and how they use this perspective to evaluate their own strategy, it is necessary to represent the neural processes that are used in evaluating multiple different strategic alternatives that both players might consider, as illustrated in Figure 5. In order to do so, the following borrows significantly from the economic game theory literature in a similar fashion to that of Yoshida et al. (2008) but within the context of strategically complex games and using SDEs to model the underlying neural dynamics within the PFC. To begin, we identify the neural encoding of a player's strategy with levels of neural activity, a strongly favored strategy is reflected in higher levels of neural activity in an analogous fashion to other decision-making activations in other regions of the brain (Gold and Shadlen, 2001; Brown et al., 2009; Simen et al., 2009; Rorie et al., 2010). In this case 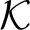
_*b*_ represents the level of neural activity associated with Black's encoding of the choice *Knight to f6* and 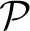
_*b*_ represents the level of neural activity associated with choice *pawn to d6*. We will also use the notation *p*(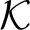
_*b*_) and *p*(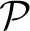
_*b*_) to represent the probabilities of black choosing each of these two moves. Either of these two choices by Black leads to different choices by White when they move next (see Figure 6) and Black needs to estimate the “value” or “weight” (greek letters in Figure 6) attributed to each of these two outcomes for Black; Black plays Knight and either White knight to *g*5 = φ_*kg*5_ or White knight to *c*3 = φ_*kc*3_, alternatively Black plays pawn and either White pawn to *c*3 = ϕ_*pc*3_ or White pawn to *h*3 = ϕ_*ph*3_. So Black also needs to encode a representation of White's likely choices, we represent the neural activity in Black's PFC associated with White's choices using labels for White's four possible moves shown in the left of Figure 6: 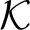
^*g*5^_*w*_ and 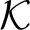
^*c*3^_*w*_ if Black plays Knight, 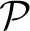
^*c*3^_*w*_ and 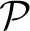
^h3^_w_ if Black plays pawn. With this interpretation we can represent changes in the level of neural activity for each move's neural encoding as a function of the other player's strategy with some statistical variation: 
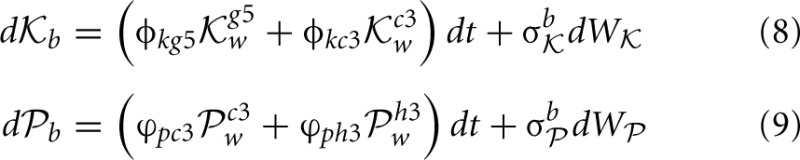
 These are a pair of Ornstein-Uhlenbeck (drift diffusion) equations in which the drift terms are not constant as they depend on other dynamic variables, in this case the levels of neural activity 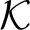
^*g*5^_*w*_, 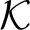
^*c*3^_*w*_, 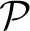
^*c*3^_*w*_, and 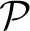
^h3^_w_, discussed shortly. Looking at the rate of change in neural activity for Black's Knight move *d*
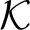
_*b*_ it is composed of a weighted sum (the weights are the constants φ_*kg*5_ and φ_*kc*3_) of the current level of activity of Black's neural encoding of White's options of two different Knight moves. Just as in Equation (1) there are noise terms σ^b^_
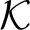
_ and σ^b^_
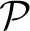
_ representing non-systematic errors in the encoding of the strategies. Both *d*
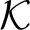
_*b*_ and *d*
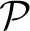
_*b*_ are independent of each other in that the level of activity of one variable does not influence the other (neither term appears in the expression for the other). The four weights that appear in Equations (8, 9) (φ_*kg*5_, φ_*kc*3_, ϕ_*pc*3_, and ϕ_*ph*3_) are based upon Black's previous experience of their own ability in playing these two strategies. For example Black believes they play 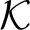
_*b*_ with strength φ_*kg*5_ against White's 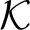
^*g*5^_*w*_ and with strength φ_*kc*3_ against White's 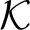
^*c*3^_*w*_. These are subjective estimates a player has developed through experience and are subject to uncertainty in there estimation, particularly when the strategies in question are unfamiliar or the other player's ability is unknown.

The Black player arrives at the decision to play either the Knight or the pawn when the absolute value of either 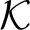
_*b*_ or 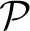
_*b*_ reaches a certain threshold value thereby signaling a decision (in principle similar to Figure 1, but see Bogacz et al. (2006) for details) and this signal cascades from the anterior PFC to the posterior PFC where this first move in Black's strategy is then turned into a motor plan by the PM cortex. The rate at which the neural activity of either 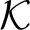
_*b*_ or 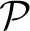
_*b*_ reaches this threshold depends on the fixed weights and the neural activity associated with White's strategy. The fixed weights can be attributed to learned and reinforced behaviors and so do not change during the time-course of a single decision, but note that these feedback (reward) based weights need to be correctly attributed to each player, so the neural encoding needs to reflect which player did what and what each player received as feedback. Incorrectly attributing feedback to the actions of different players will result in misattributing the weights associated with each player's strategy in future games. The neural activity associated with White's choices is a dynamic quantity associated with Black's representation of what Black believes White will choose to do after Black has made their move. This requires Black to represent White's decision-making process and White will choose the strategy that best advantages them given what White thinks Black will do following White's move. Just looking at *d*
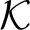
_*b*_ above, Black needs to encode the following decision-making processes in order to accurately represent what White is likely to do next: 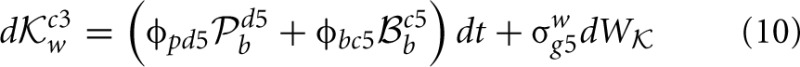 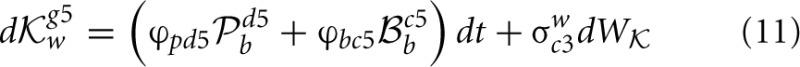 Note that White's move of either 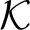
^*g*5^_*w*_ or 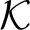
^*c*3^_*w*_ have fixed weights on the right hand side of Equations (10, 11) representing the learned payoffs White has for Black's moves of pawn to *d*5 and Bishop to *c*5, but while there are only two Black moves in Equations (10, 11) (see the caption to Figure 5), the weights White attributes to these two possible choices of Black's are different because White playing Knight to *g*5 first is strategically different to White playing pawn to *c*3 first, hence the payoff weights for White are different and this has a follow-on effect in Black selecting a move in Equations (8, 9). In this sense Black has an internal representation that is encoded in their neural activity of how White will make a decision, and this in turn depends on what Black will do in response to White's choices. Also note that Black needs to be able to encode the payoffs to White in order to estimate White's possible choices, so Black needs to distinguish between their payoffs and White's payoffs.

In such a situation it is not obvious that there is a solution to these dynamics that allow Black to settle on a decision as to which is the best move to make. Fortunately these types of SDEs have known solutions, particularly in the case where the drift term, that which appears immediately before the *dt* in Equations (8), (9), (10), and (11), is linear in the dynamic variables (Plastino and Plastino, 1998). These solutions take the form of probability distributions over the strategies that Black has available to them: 
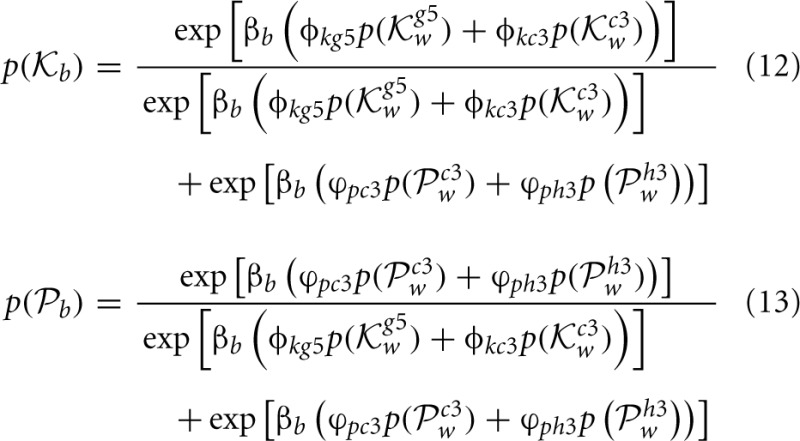
 where *p*(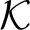
^*g*5^_*w*_) and *p*(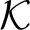
^*c*3^_*w*_) are probability distributions defined in an analogous fashion as Equations (12, 13) but for the White player and a similar substitution for β_*b*_ as for Equation (7) has been made in order to simplify the expression. Both *p*(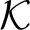
^*g*5^_*w*_) and *p*(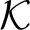
^*c*3^_*w*_) have a further dependence on the probabilities over Black's subsequent move choices after White's next move. Note that although Equation (7), (12), and (13) describe entirely different cognitive processes they have a very similar functional form:

(14) $$p(x_{i})=\frac{\exp[\beta f_{i}(c,x)]}{\sum_{k}\exp[\beta f_{k}(c,x)]}$$

in which β is a noise parameter, *f*_*i*_(*c,x*) are linear functions of constants *c*_*j*_ and dynamic variables *x*_*i*_ represented here by a vector of such terms *c* and *x*. Such exponential forms of probability distributions over choices are common in models of bounded rationality in economics (McKelvey and Palfrey, 1995; Wolpert et al., 2011), in computational models of simple reinforcement learning (Williams, 1992; Sato et al., 2002) and as models of neural activity in theoretical psychology (Bogacz et al., 2006; McMillen and Holmes, 2006). However, to date the connection between the generic dynamics of SDEs, theoretical neuropsychology and expertise does not appear to have been made.

## 4. Discussion

The need to distinguish between that which motivates a person's choices and that which motivates another person's choices, and how these motivations interact in a single individual's decision making processes is a critical component to the way in which we interact socially. How such strategic considerations are then integrated with our perceptual understanding of the environment is a challenging question that can be discussed in terms of skill in competitive board games.

This article has developed a model of visual perception and opponent modeling within the framework of SDEs representing task specific neural activity and subsequent decision-making. The modeling of game-scene perception follows a well developed research paradigm of progressively more sophisticated representations of the scene culminating in the most complex representations that a player's experience allows for. In the highest ranking experts this can result in an initial, rapid perception of the board being sufficient to initiate a single good move almost immediately after being presented with a game. More generally this expert perception activates the eyes to rapidly and efficiently search the board for the most likely candidate regions and base moves from which to explore possible branches of play. Once one of a small set of good base moves has been selected an expert is able to search forward in the game tree with greater strategic depth than a non-expert and to be able to more effectively estimate the likely replies of their opponent and how these replies influence the player's choice of the next move to make. While these general psychological results have existed for some time now, there appears to be no previous analysis of how SDEs, their dynamics and probabilistic outcomes in terms of neural activity are related to the psychological literature of expertise.

The formal representation of how a player might model their opponent's state of mind, particularly their strategy space, incentives, constraints, and the influence these aspects have on their decisions, is a challenging task. However, the formal techniques have been available for some time and the results in terms of probability distributions are not particularly divergent from some previously established theoretical models. The demanding task is in the integration of the vast amount of data available from the neuro-imaging literature into a coherent whole that is both consistent and convincing. Theoretical (Yoshida et al., 2008) and empirical (Bhatt and Camerer, 2005) arguments for a game theoretical basis of a ToM have appeared in the literature, but these have not previously been extended to more complex tasks, expertise or used SDEs as the basis for their modeling. Furthermore, the relationship to the broader neuroscience literature has had almost no coverage in this respect, specifically how the different neural networks and their functional roles might be integrated as a whole in the modeling of expertise. The critical difference in the approach put forward here is that, unlike previous neural-connectionist-reinforcement paradigms, this model represents a player's internal representation of the other player's internal strategic state of mind, not just their own. This entails several important cognitive steps, principally recognizing that the task environment contains another cognisant entity that will dynamically adapt their choices according to their beliefs or expectations about the choices others will make. It also requires the motivations and constraints of this other entity to be internally represented and so we need to consider how our ToM and reward mechanisms interact with our strategic perspective (and how we model the strategic perspective of others). This represents a significant step in showing how different cognitive processes might be integrated to help explain some of the prodigious skills we are all capable of expressing to some extent, and the role these skills might play in a broader context, such as our everyday social lives.

## Funding

This work was supported in part by US AirForce Grant AOARD 104116.

### Conflict of interest statement

The author declares that the research was conducted in the absence of any commercial or financial relationships that could be construed as a potential conflict of interest.
